# Cannabidiol and *Cannabis Sativa* as a potential treatment in vitro prostate cancer cells silenced with RBBp6 and PC3 xenograft

**DOI:** 10.1007/s11033-022-08197-0

**Published:** 2023-02-28

**Authors:** Lesetja R. Motadi, Zodwa E. Jantjies, Boitumelo Moleya

**Affiliations:** Department of Biochemistry, University of Johannesburg, Auckland Park Campus, Johannesburg, South Africa

**Keywords:** Cisplatin, Cannabidiol, Cannabis sativa extract, siRBBP6, And apoptosis

## Abstract

**Background:**

Prostate cancer is the second most frequently occurring carcinoma in males worldwide and one of the leading causes of death in men around the world. Recent studies estimate that over 1.4 million males are diagnosed with prostate cancer on an annual basis, with approximately 375,000 succumbing to the disease annually. With current treatments continuing to show severe side effects, there is a need for new treatments. In this study we looked at the effect of *cannabis sativa* extract, cannabidiol and cisplatin on prostate cancer cells, PC3.

**Methods:**

In addressing the above questions, we employed the MTT assay to measure the antiproliferative effect on PC3 cells following treatment with varying concentrations of *Cannabis sativa* extract, cisplatin and cannabidiol. xCELLigence was also used to confirm the IC50 activity in which cells were grown in a 16 well plate coated with gold and monitor cell attachment. Caspase 3/7 activity was also measured using 96 well-plate following treatment. Western-blot and qRT-PCR was also used to measure the gene expression of tumour suppressor genes, p53, Bax and Bcl2. Animal studies were employed to measure the growth of PC3-mouse derived cancer to evaluate the effect of compounds in vivo.

**Results:**

From the treatment with varying concentrations of *Cannabis sativa* extract, cannabidiol and cisplatin, we have observed that the three compounds induced antiproliferation of PC3 cancer cell lines through the activation of caspase 3/7 activity. We also observed induction of apoptosis in these cells following silencing of retinoblastoma binding protein 6 (RBBP6), with upregulation of p53 and bax mRNA expression, and a reduction in Bcl2 gene expression. The growth of tumours in the mouse models were reduced following treatment with cisplatin and cannabidiol.

**Conclusion:**

We demonstrated that cannabidiol is a viable therapy to treat prostate cancer cells, in combination with silencing of RBBP6. This suggests that cannabidiol rather *Cannabis sativa* extract may play an important role in reducing cancer progression.

## Introduction

Prostate cancer is one of the major causes of death among men worldwide. Currently, prostate cancer accounts for 14% of all male malignancies, it is the most diagnosed cancer in men in over one-half of the world's countries and is second to lung cancer as the most frequently occurring cancer in men worldwide. Prostate cancer was the fifth leading cause of cancer-related deaths in men worldwide in the year 2020. Even though the exact origin of prostate cancer is unknown, several factors, including age, family history, lifestyle factors, and testosterone levels, are linked to a higher chance of acquiring the disease [1–3].

Cannabinoids are naturally occurring compounds found in the *Cannabis sativa* plant and are classified into three categories based on their origin: plant-derived cannabinoids such as ∆9- tetrahydrocannabinol (∆9-THC), endocannabinoids and synthetic molecules that mimic the structure of either plant or mammalian cannabinoids. More than 120 phytocannabinoid entities are found in cannabis plant extracts, with the main clinical interest being cannabidiol (CBD) and tetrahydrocannabinol (THC) [4–7]. In recent years, cannabinoids have gained more interest in treating side effects associated with chemotherapy, such as nausea. There is also growing interest in the anti- cancer potential of *Cannabis sativa* secondary metabolites. However, there is little data to support their use or effectiveness in cancer treatment. Research has demonstrated that cannabinoids possess some anti-cancer properties since the identification of cannabinoid receptor type 1 (CB1) and cannabinoid receptor type 2 (CB2) [8, 9]. Recent studies have demonstrated that there is a possibility to cultivate *Cannabis sativa* that is CBD-biased, and this also depends on the geographical impact from where the plant originated^11^. CBD has been found to elicit anti-proliferative and apoptotic effects in breast cancer cell lines, MDA-MB 321 and MDA-MB 436 [10, 11]. Several studies have acknowledged cannabidiol possesses anti-cancer properties against several cancers, but none has reported their effect with co-treatment with other cancer therapies [12, 13]. Despite the lack of a described and understood molecular mechanism of action, CBD is still the most effective cannabinoid for use in developing anti-cancer drugs [14].

Researchers have resorted to animal tumor models as they are a reliable predictor in preclinical cancer research, given that they can produce reliable tumor growth. Although many studies have examined the impact of cannabis on prostate cancer in cell culture experiments, very few papers have established if these effects occur in in vivo animal models. Few publications have determined whether these effects occur in in vivo animal models, regardless of the fact that several studies have explored the efficacy of cannabinoids on prostate cancer in cell culture studies [15]. Numerous studies have been done on CBD concerning various cancers, including prostate and lung cancer [16]. Some of these studies discovered that the extracts of cannabis enhanced with CBD effectively reduced the growth of tumors in androgen receptor- positive LNCaP xenografts but amplified tumor growth in androgen receptor-negative DU-145 xenografts. Correspondingly, to determine the effects of CBD in vivo, the xenograft mouse models [17]. The migration/invasion and viability of the HNSCC cells have been drastically reduced in a dose- and time-dependent manner [18, 19]. In other studies, unrelated to cancer, CBD did not affect heart rate or blood pressure under normal conditions, but in animal stress models, CBD reduces heart rate and blood pressure [5, 20, 21]. Results from studies conducted on humans and animals interpret/illustrate that CBD possesses extremely different effects compared to THC [22, 23]. Although these studies show cannabinoids have the potential to target prostate cancer, there is still the need to implement more research to conclusively establish the efficacy of cannabinoids in vivo and the importance of co- treatment with either gene therapy or chemotherapy.

In a number of malignancies, including oesophageal cancer [24], breast cancer [25], lung cancer [26], and cervical cancer [27], retinoblastoma-binding protein 6 (RBBP6), which regulates cell proliferation, cell cycle, and cell apoptosis, is greatly up-regulated and is associated with poor clinical prognosis. Cell cycle arrest, a characteristic of carcinogenesis that is caused by RBBP6 overexpression, is significantly linked to the growth of cervical and oesophageal cancer tumors [4, 9]. This, therefore, suggests that RBBP6 may serve a critical role in the malignant phenotype of human cancer [4, 10, 11]. In addition to this, the results indicated that RBBP6 may promote tumorigenesis by increasing cancer cell proliferation. In this study, we investigated the anti-cancer properties of *Cannabis sativa* extract, CBD and cisplatin on prostate cancer cells, PC3, in combination with siRBBP6 gene therapy.

## Materials and methods

### Cell culture and RNA interference

American Type Culture Collection was used to obtain the PC3 cell lines (Manassas, VA, USA). These cells were regularly grown in RPMI 1640 media (Hyclone®; Thermo Fisher Scientific Inc, Waltham, MA, USA) with 5% of FBS (fetal bovine serum) and 100 U/mL penicillin at 37 °C in a humidified environment with 5% CO2. We bought MRC5 and Hek293 from (ATCC, USA). Natural Therapy (Cape Town, South Africa) provided the complete *Cannabis sativa* extract, and Sigma provided the CBD and cisplatin.

Using GeneSilencer siRNA Transfection Reagent (Genlantis, San Diego, CA, USA), cells were transfected for RNA interference studies with 75 nM RBBP6 siRNA (Ambion) or control siRNA (scrambled siRNA, a universal negative control). Real-time quantitative PCR (qPCR) and western blot analysis were used to assess the effectiveness of siRNA-mediated RBBP6 knockdown 48 h after transfection.

### Cell proliferation

By the use of 3-(4,5-dimethylthiazol- 2-yl)-2,5 diphenyltetrazolium bromide (MTT) assay, the proliferation of cells was assessed. Cells (3 × 10^3^) were cultured in the 96-well culture plates and treated with either 30 µM *Cannabis sativa* extract, 10 µM CBD, 3 µM cisplatin, or transiently transfected RBBP6 siRNA, then co-treated with either CBD or cisplatin. Following treatment, 5 mg/mL of MTT was added to each well in culture media, and each well was then incubated at 37 °C for 4 h. The reaction was stopped by adding 150 µl of dimethyl sulfoxide (DMSO) to dissolve the formazan product. The micro ELISA plate reader was read at a wavelength of 570 nm. There were at least three runs of each assay.

### Detection of caspase 3/7 activity

Following the manufacturer’s instructions, a colorimetric method was used for measuring the activity of Caspase 3/7 (Caspase-Glo® 3/7 Assay kit: Promega Corp, Fitchburg, WI, USA). Briefly, 30 µM *Cannabis sativa* extract, 10 µM CBD, 3 µM cisplatin, or transiently transfected RBBP6 siRNA were applied to 5 × 10^4^ cells planted in 96-well plates for 24 h, followed by 48 h of co-treatment with either CBD or cisplatin. The cell lysate was then combined for an hour at room temperature with Caspase-glo 3/7 reagents that had been equilibrated. A GloMax 96 luminometer was used to measure luminescence (Promega Corp).

### Flow-cytometry

Suspension cultures were prepared from PC3 cells following different treatments as previously described. Cells were incubated with annexin V and propidium iodide for 60 min (1 h) and then later fixed with 2% of PFA (paraformaldehyde) and acquired on a BD FACSCalibur™ Flow Cytometer and analyzed using FlowJo software. The MetaMorph Offline Premier Software (Molecu lar Devices, Sunnyvale, California) was used to acquire Fluorescence images.

### RT q-PCR and reverse-transcriptase PCR (RT-PCR)

Trizol® reagent (Invitrogen) coupled with DNase digestion step, with the Real Star Kit (Durviz, Valencia, Spain), was used to isolate RNA. The RNA was further used to form the complementary DNA strand (reverse transcription) using the First Strand cDNA Synthesis Kit (Promega) to form cDNA. Applied Biosystems provided TaqMan probes (Foster City, CA). A 7900 Real-time PCR System was used to carry out the amplification (Applied Biosystems). Using 18S RNA levels as a benchmark, each value was modified.

### Western blot analysis

Cells were treated as specified and harvested, and the total protein concentration in the supernatant was assessed using the Bramhall test to quantify protein expression. Proteins were electroblotted onto polyvinylidene fluoride membranes after cell lysates were resolved on polyacrylamide gels with a 4–8 percent SDS content. Secondary antibodies were used in conjunction with the main antibodies anti-p53, anti-RBBP6, and anti-GAPDH (Abcam) to incubate the blots. The Amersham Enhanced Chemiluminescence Detection Kit (GE Healthcare, Uppsala, Sweden) was used to obtain luminograms, and Quantity One software was used for densitometric analysis (Bio-Rad).

### Mouse models

All animal procedures were conducted according to the guidelines and approval of the North-West University Animal Care Committee NWU-00443-21. Immunodeficient 8-week-old nude mice were obtained from the NWU animal unit, and tumors were generated through subcutaneous (s.c.) injection of 10 × 10^6^ PC3 cells in phosphate buffered saline solution (PBS) with added 0.1% glucose. Vernier callipers were used every day to measure tumors frequently. Once the average size of 100 mm^3^ was acquired, the animals were randomly allocated to various groups and inoculated peritumorally for 10 days with either CBD (150 mg/kg/d), cisplatin (50 mg/kg/d), *Cannabis sativa* extract (200 mg/kg/d) or vehicle in 100 μl of PBS supplemented with 5 mg/mL defatted and dialyzed bovine serum albumin (BSA). Animals have unlimited access to food and water ad libitum. During this time, tumors were regularly measured, and the volume was computed as (4π/3) × (width/2)^2^ × (length/2). At the end of the treatment phase the mice euthanized and tumors were collected for western blot and qPCR analysis.

### Statistical analysis

All data were reported as mean ± standard deviation, and the significance levels were calculated using the student’s *t*-test and one-way analysis of variance (ANOVA) analysis. Statistical significance was defined as a P-value of 0.05 or 0.01 or less. Software called SPSS/Win11.0 was used for the statistical analysis.

## Results

### Cell viability and anti-proliferation of prostate cancer cell lines

**Fig. 1:**
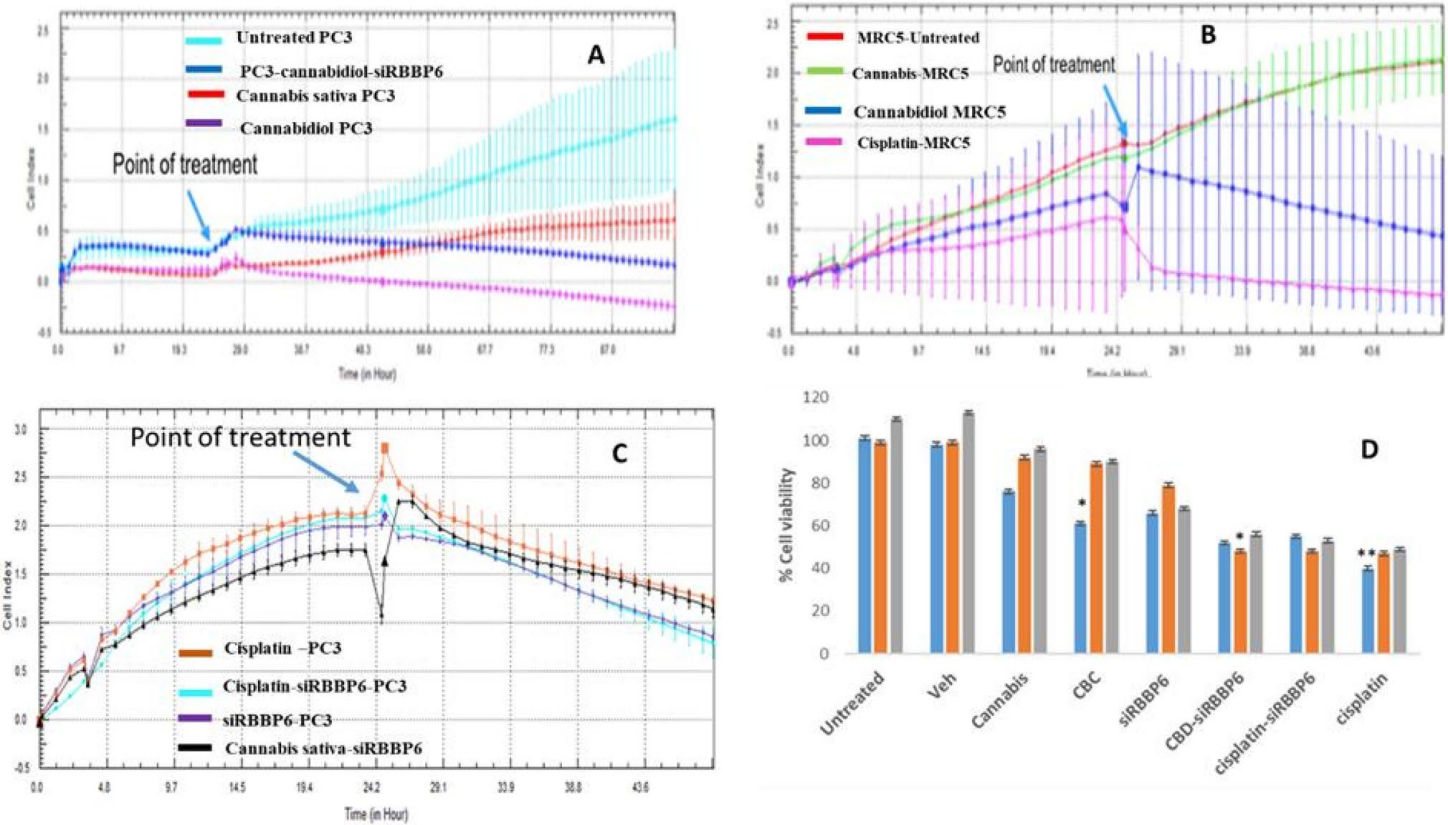
xCELLigence and MTT assay results. CBD inhibits the proliferation of prostate cancer cells. PC3, MRC5, Hek 293 cells were treated with DMSO 0.05% (100% of DMSO was previously dissolved in media to make 0.05%), 30 µM *Cannabis sativa* extract (10 mg of crude previously dissolved in 1 ml of 100% DMSO to make final concentration of 10 mg/ml), CBD 10 μM, siRBBP6, 10 µM CBD-siRBBP6, 6 µM cisplatin-siRBBP6 and 6 µM cisplatin for 32 h and then subjected to MTT assay. **A, B, C** are xCELLigence live tracking of cell proliferation of cells following treatment with *Cannabis sativa* extract, CBD, cisplatin, and siRBBP6. **D** is the MTT assay analysis. The data represent the mean ± SD of three independent experiments. *P < 0.05, **P < 0.01, and ***P < 0.001 compared with the control group

AnAn unregulated proliferation of cells is an essential characteristic of the survival of cancer cells and leads to the formation of aggressive tumors. As shown in previous studies [14], RBBP6 is highly expressed in cancer cells and plays a major role in cell proliferation. Therefore, we performed the MTT assay to evaluate the effect of *Cannabis sativa* extract and CBD on prostate cancer cell proliferation in combination with RBBP6 silencing. We found that *Cannabis sativa* extract has no significant effect on prostate cancer cell proliferation, whereas CBD induces a strong decrease in the proliferation of PC3 cells (Fig. 1). There was minimal effect on MRC5 and Hek 293 cell lines when treated with *Cannabis sativa* extract and CBD. However, there was a strong reduction of cell proliferation in siRBBP6-transfected cells concomitantly treated with CBD in MRC5 and Hek-293 cell lines. For further studies, we chose lower concentrations for *Cannabis sativa* (30 µM) and CBD (10 μM) because they have a lower direct effect on proliferation. We tested the anti-proliferative ability of low doses of cisplatin in the absence of RBBP6. As shown in Fig. 1C and D, cisplatin significantly inhibits siRBBP6- induced proliferation in PC3 cells. With xCELLigence, we were able to confirm that CBD and cisplatin have an inhibitory effect in PC3 cells.

## *Caspase 3/7 activity analysis following treatment with *Cannabis sativa *extract, CBD and siRBBP6*

**Fig. 2:**
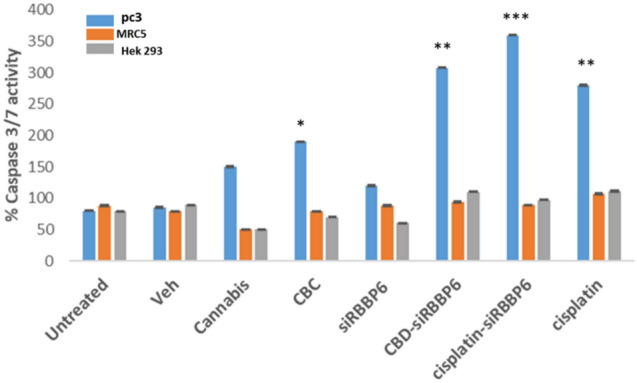
Caspase 3/7 assay of silencing of RBBP6 in PC3 cells, with co-treatment with CBD or cisplatin induces caspase 3 mediated apoptosis. Relative luminescence expression of caspase3/7 in PC3, MRC5 and Hek 293 cells treated with 30 µM *Cannabis sativa* extract, 10 µM CBD, cisplatin and siRBBP6 for 48 h. The data represent the mean ± SD of three independent experiments. *P < 0.05, **P < 0.01 and ***P < 0.001 compared with the control group

The crucial apoptotic executor caspase-3 must be activated for DNA fragmentation and the usual morphological alterations of apoptotic cells. To determine whether or not the combination of CBD, siRBBP6, and *Cannabis sativa* extract causes apoptosis mediated by caspase-3/7 activation, we measured the bioluminescent intensities of the treated cells to assess the caspase-3/7 activity. The activity of caspase 3/7 was significantly activated in PC3 cells treated with all the described treatments and their combinations (Fig. 2). Both CBD-siRBBP6 and cisplatin-siRBBP6 cells showed the most increase in caspase 3/7 activity, suggesting concomitant transfection with siRBBP6 and cisplatin or CBD treatment might be more effective than a single treatment.

### Apoptosis analysis following treatment with CBD

**Fig. 3:**
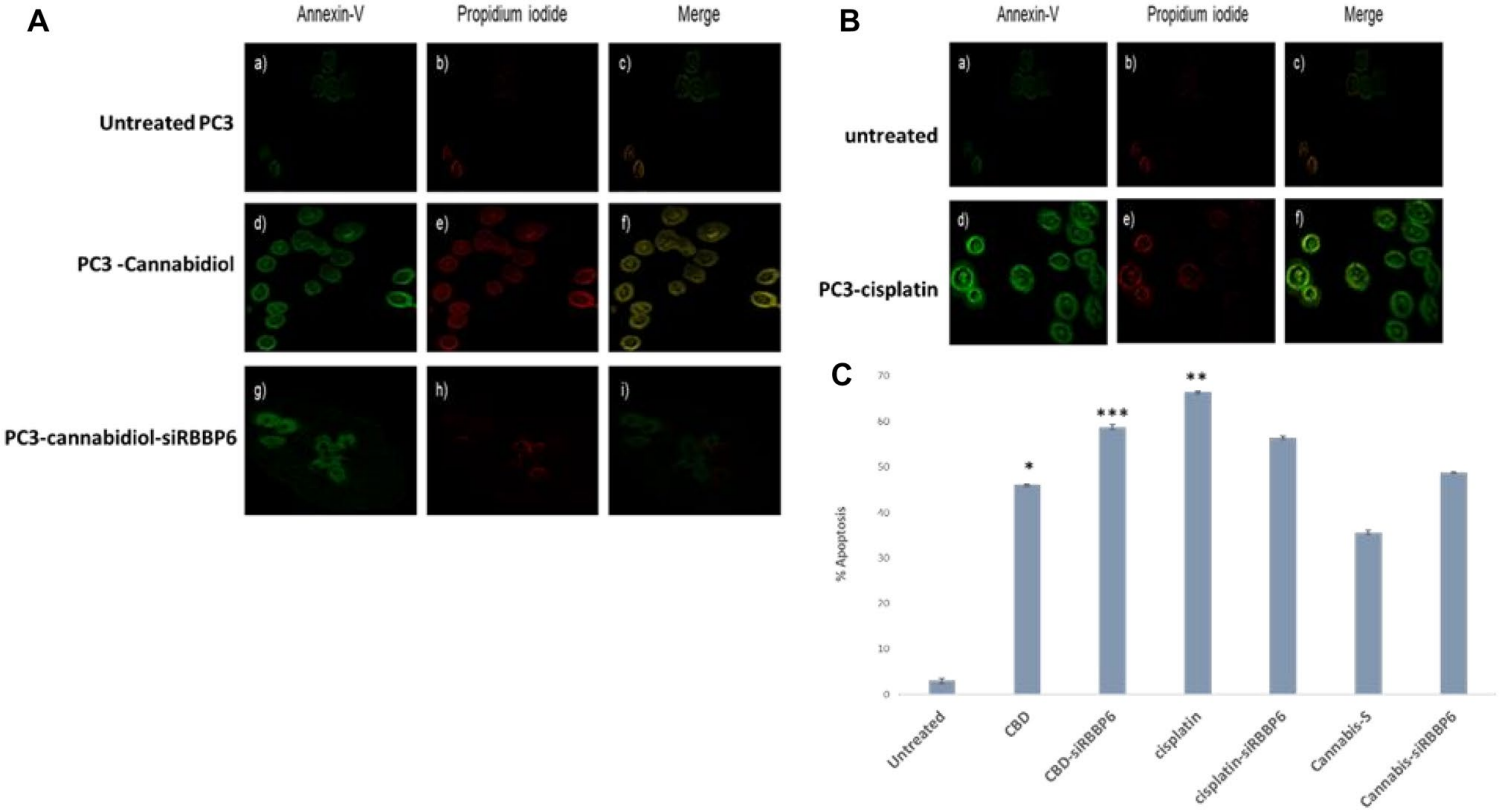
Morphological analysis of apoptosis in PC3 cells transfected with siRBBP6 and co- treated with CBD and cisplatin. PC3 cells were treated with CBD, CBD-siRBBP6, cisplatin and cisplatin-siRBBP6 for 48 h. **A** morphological apoptosis analysis of PC3 treated with CBD and CBD-siRBBP6 **B** morphological apoptosis analysis of PC3 treated with cisplatin and cisplatin- siRBBP6 **C** PC3 cells apoptosis analysis determined by flow cytometry. Results are expressed as the mean ± SEM of three independent experiments. *P < 0.05, **P < 0.01, and ***P < 0.001 vs. untreated

We investigated and calculated the percentage of apoptotic cells after CBD and cisplatin treatment to see if these agents' growth of PC3 cells was inhibited because of enhanced apoptosis. The outcomes demonstrated that CBD boosts PC3 cells' rate of apoptosis In particular, the percentage of apoptotic cells after 48 h of treatment with 10 μM CBD and 10 μM- siRBBP6 was 56.5 ± 3.25% (*P *< 0.05) and CBD-siRBBP6 58.7 ± 2.76% (*P *< 0.001), respectively. This was significantly higher than the percentage of control cells (3.00 ± 2.98%). With cisplatin, similar results were observed with increased apoptosis following treatment of PC3 cells with 3 μM cisplatin for 48 h; the percentage of apoptotic cells following treatment with 3 μM cisplatin and 3 μM-siRBBP6 for 48 h resulted in 66.3 ± 2.67% (*P *< 0.01) and in cisplatin-siRBBP6 56.4 ± 3.33% apoptotic cells, respectively, which was significantly greater than that in control cells (3.00 ± 2.98%). However, *Cannabis sativa* extract has no significant effect on the apoptosis rate of PC3 cells (3C) Morphological analysis was also shown to support the results as shown by flow cytometry (Fig. 3).

### Gene expression analysis

**Fig. 4:**
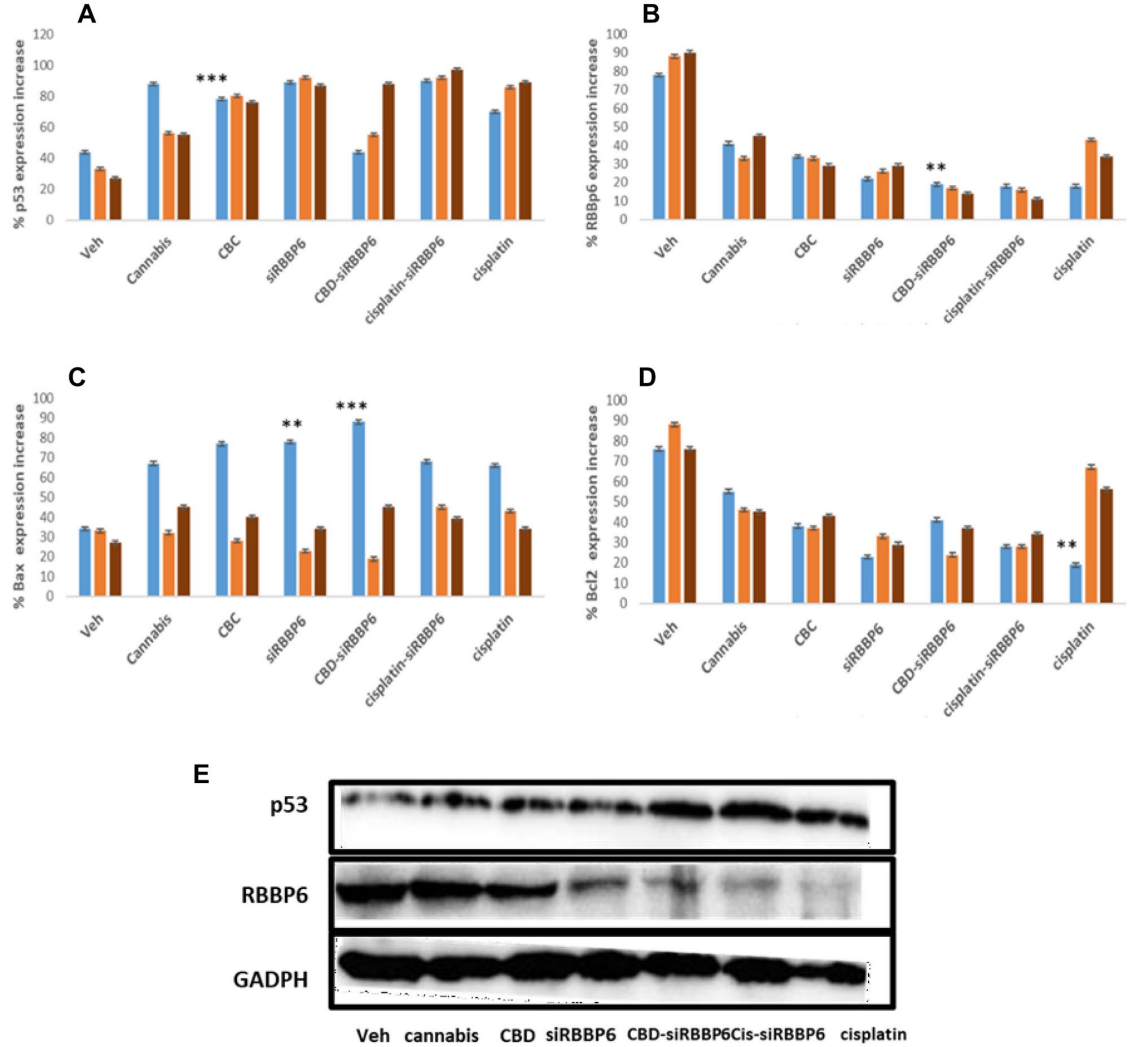
The expression analysis of apoptotic genes p53, Bax, Bcl2 and RBBP6 in PC3, MRC5 and Hek 293 cells treated with 30 µM *Cannabis sativa* extract, 10 µM CBD, 3 µM Cisplatin and siRBBP6 for 48 h. Following treatment, gene expression analysis was conducted by qRT-PCR and western blot. **A–D** levels of mRNA of p53, Bcl-2, Bax and RBBP6 and **E** protein levels in PC3 cells

Our next step was identifying genes that could play a role in the antitumoral effect of CBD and cisplatin. Through the comparison of the mRNA expression profiles of p53, Bax, bcl2 and RBBP6 following treatment with 30 µM *Cannabis sativa* extract, 10 µM CBD, 3 µM Cisplatin and siRBBP6 for 48 h. Incubation with 10 µM CBD resulted in a parallel increase in p53, and Bax mRNA levels by 60% and 70%, respectively (Fig. 4A and C). In addition, co-transfection with siRBBP6 also significantly increased the mRNA expression levels of Bax by 81%. Observing the mRNA expression of an anti-apoptotic gene, Bcl2, following treatment with 30 µM *Cannabis sativa* extract, 10 µM CBD, 3 µM Cisplatin and siRBBP6 for 48 h, there was a decrease, albeit not significant, in Bcl2 compared to Bax from 74 to 27% between untreated and treatment with CBD with co-transfection with siRBBP6 showing a further significant drop to about 11% (Fig. 4B). With all siRBBP6-related transfections, RBBP6 mRNA and protein were all down-regulated. The p53 protein was highly expressed in all CBD, CBD-siRBBP6, cisplatin and cisplatin-siRBBP6 treated cells (Fig. 4E).

### In vivo* evaluation of CBD*

**Fig. 5:**
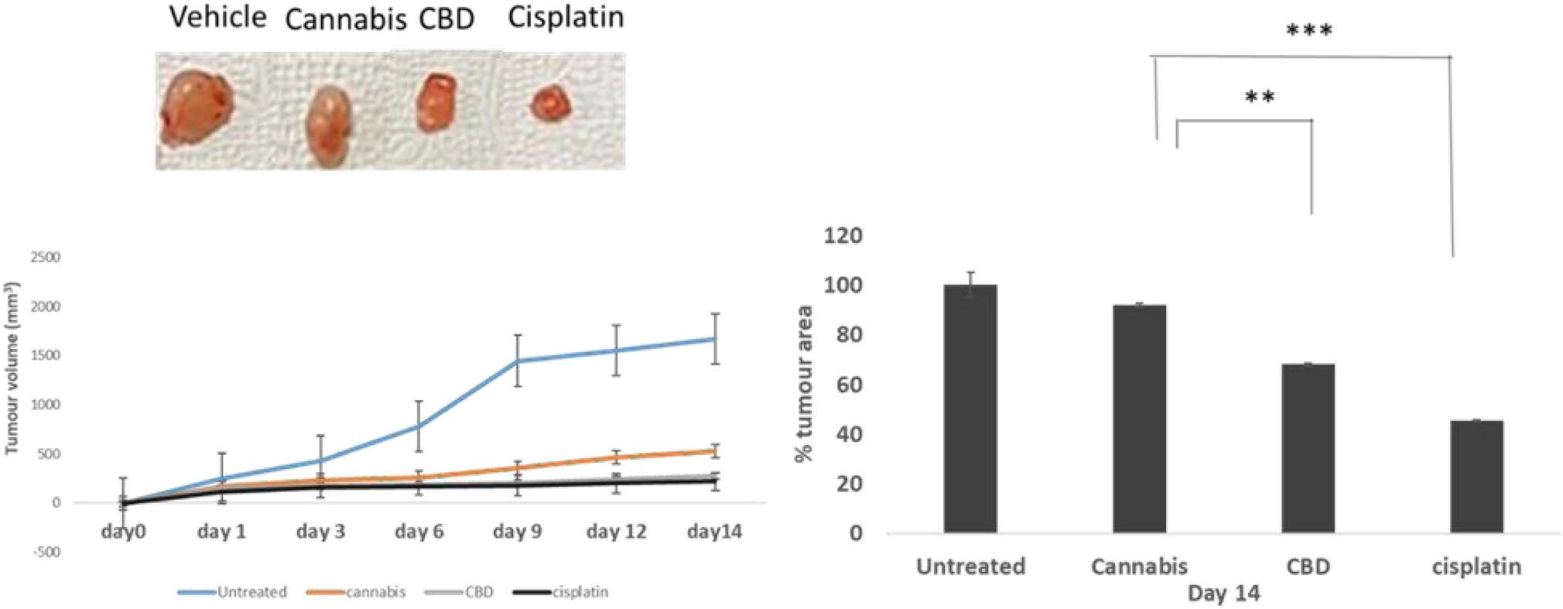
Treatment by CBD and cisplatin can reduce the progression of prostate cancer cells in vivo. When the desired size at day 0 was reached by the tumours, animals were then injected with either CBD (150 mg/kg/d), or cisplatin (50 mg/kg/d) and or *Cannabis sativa* extract (200 mg/kg/d) for 10 days (*n* = 8 for each experimental group). The volume of the tumor was measured at the indicated times. The outcomes are presented as the percentage of tumour volume growth relative to day 0. * *P *< 0.05, ** *P *< 0.01 and *** *P *< 0.001 were significantly different from untreated tumours on the corresponding day of treatment. Photographs of a representative untreated, *Cannabis sativa* extract, CBD, and cisplatin

This was followed by further validating the anti-tumour capacity of *Cannabis sativa* extract, CBD and cisplatin using a xenograft mouse model. About 2 × 10^6^ of PC3 cells were injected subcutaneously of BALB/c nude mice to generate PC3xenograft mouse models. As shown in Fig. 5, xenograft tumour sizes were meaningfully reduced in mice treated with CBD and cisplatin in comparison with those in untreated control mice and *Cannabis sativa* extract-treated mice. These results indicate inhibited growth of PC3 cells (in vivo) by CBD and cisplatin. Furthermore, mice that received either CBD or cisplatin were observed to have notable inhibitions of tumour growth and weight as compared to those that received *Cannabis sativa* extract, which means that CBD rather than the plant extracts of *Cannabis sativa* can be used as part of combinational therapy against prostate cancer.

## Discussion

Behind lung cancer, prostate cancer is the second most common cancer among men. According to studies, roughly 14% of men will experience the condition at some point in their lives. Prostate cancer is also the seventh most lethal malignancy in men globally [28–30]. Depending on the stage of the disease, many treatments are available for prostate cancer. For instance, radiation therapy might be used to treat stage 1 prostate cancer. However, stage three patients could also need hormone therapy and external radiation [24]. Depending on the stage of the disease, many treatments are available for prostate cancer. For instance, radiation therapy might be used to treat stage 1 prostate cancer. Although in stage three, patients might also need hormone therapy and external radiation [25]. Most treatments for localized prostate cancer work well; however, there are discussions regarding *Cannabis sativa* extract, THC and CBD as a natural alternative to prostate cancer. In this study, we looked at the effect of *Cannabis sativa* extract and CBD with some silencing of RBBP6 as co-treatment [13].

In other studies, the CBD compound has been found to be cytotoxic and inhibit tumour growth and induce apoptosis in glioma cells [26]. Due to their capacity to control pain as well as their anti-inflammatory, suppression of growth of cells, and apoptotic proper ties, cannabinoids have been employed in cancer therapy [27]. The current work looked at the molecular underpinnings of CBD’s anti-tumor efficacy and *Cannabis sativa* extract. We demonstrated that both *Cannabis sativa* extract and CBD prevent the growth of prostate cancer; however, it is CBD, not the entire extract, that does so. We made the first known finding that CBD could prevent prostate cancer growth both in vitro and in vivo. We also demonstrated for the first time that CBD, in combination with silencing of RBBP6, a negative regulator of p53, has the capacity to suppress the growth of prostate cancer [31–33].

It is crucial to continue finding drug treatments that can target this axis in order to prevent cancer growth and metastasis caused by altered or inactive p53 pathways, particularly in patients with prostate cancer. This study demonstrated that CBD inhibits prostate cancer cells’ activation of Bcl2 signalling transduction pathways in this study. Two essential molecules (Bax and Bak), which are significant apoptosis inducers, are involved in these pathways. This further explains how CBD can prevent the growth of prostate cancer [32, 34]. This observation is particularly significant because it is widely known that RBBP6 overexpression is linked to cancer growth and p53 inactivation, both of which have detrimental effects on prognosis, particularly in prostate cancer [26]. We demonstrated that CBD produces cytotoxicity in the human prostate cancer cell line PC3 but not in healthy MRC 5 and Hek 293 cell lines. CBD has a palliative effect and is less toxic to normal cells than other natural compounds. In various cancer cell lines, including breast, colorectal, leukaemia, and pancreatic tumor cells [9], it is known that CBD triggers apoptosis-mediated cell death. We noted that CBD affects prostate cancer cells’ ability to commit to apoptosis. Additionally, we discovered that CBD treatment of PC3 cells raises levels of caspase 3/7 activity, indicating that the lethal effect of CBD on prostate cancer cells is linked to apoptosis. We did not explore the specific cell death mechanism induced by CBD treatment. We demonstrated that both Bax and p53 are up-regulated in these cells and that the Bax/Bcl2 ratio increases, suggesting that Bax and Bcl2 may be involved in the mechanism of CBD-mediated cell death in prostate cancer cells.

The present report shows that cisplatin induces apoptosis of prostate cancer cell lines in vitro and exerts a remarkable growth-inhibiting effect in models of PC3 prostate cancer in vivo. Moreover, these findings also show that cisplatin exerts a potent inhibitory effect on spreading prostate cancer cells to adjacent locations, hence suggesting that cisplatin may also the ability to decrease the proliferation of prostate cancer cells. To inhibit cancer cells proliferation, thus effectively killing them, biotherapies such as gene targeting and anti-cancer drug cocktails using chemicals are recently one of the most suggested therapies for anti-cancer chemotherapy [35, 36]. In this study, we discovered that cisplatin, a cytotoxic medicine for prostate cancer, has anti-cancer potential. When coupled with RBBP6 silencing, cisplatin may considerably reduce the development of cancer cells compared to therapy with either drug alone. Patients with cancer who get chemotherapy may experience a number of side effects, including fatigue, nausea, hair loss, and diarrhea due to harm to normal cells. CBD, on the other hand, has a palliative effect and is less toxic to healthy cells. Our results show that cisplatin induces cytotoxicity in human prostate cancer cell lines and on the normal MRC 5 and Hek 293 but not in normal MRC 5 and Hek 293 cell lines. Consequently, the coupled treatment with siRBBP6 can efficiently lessen severe side effects by reducing toxic chemotherapeutic drugs in cancer patients during treatment.

## Conclusions

In conclusion, these results further suggest that CBD is an effective anti-tumor drug which possesses anti-proliferative and pro-apoptotic properties. Additionally, these findings point to a crosstalk between RBBP6 silencing and CBD treatment rather than *Cannabis sativa* extract. Moreover, CBD-siRBBP6 has shown an important role of p53 up-regulation in prostate cancer, a tumor microenvironment modulating property. In conclusion, the findings of this study promote using CBD in cancer patients mostly with an inactivated p53 gene.

## Data Availability

The data used and analyzed in this study are available from the Corresponding author on reasonable request.
